# Interaction between contrasting rice genotypes and soil physical conditions induced by hydraulic stresses typical of alternate wetting and drying irrigation of soil

**DOI:** 10.1007/s11104-018-3715-5

**Published:** 2018-06-29

**Authors:** Huan Fang, Hu Zhou, Gareth J. Norton, Adam H. Price, Annette C. Raffan, Sacha J. Mooney, Xinhua Peng, Paul D. Hallett

**Affiliations:** 1 State Key Laboratory of Soil and Sustainable Agriculture, Institute of Soil Sciences, Chinese Academy of Sciences, No.71 East Beijing Road, Nanjing, 210008 China; 2 University of Chinese Academy of Sciences, No.19A Yuquan Road, Beijing, 100049 China; 3 School of Biological Sciences, University of Aberdeen, Aberdeen, AB24 3UU UK; 4 Centre for Plant Integrative Biology, School of Biosciences, University of Nottingham, Sutton Bonington Campus, Sutton Bonington, Loughborough, LE12 5RD UK

**Keywords:** Rice roots, Genotype, Macropores, Mechanical impedance, Soil structure, X-ray CT

## Abstract

**Background and aims:**

Alternate wetting and drying (AWD) saves water in paddy rice production but could influence soil physical conditions and root growth. This study investigated the interaction between contrasting rice genotypes, soil structure and mechanical impedance influenced by hydraulic stresses typical of AWD.

**Methods:**

Contrasting rice genotypes, IR64 and deeper-rooting Black Gora were grown in various soil conditions for 2 weeks. For the AWD treatments the soil was either maintained in a puddled state, equilibrated to −5 kPa (WET), or dried to −50 kPa and then rewetted at the water potential of −5 kPa (DRY-WET). There was an additional manipulated macropore structure treatment, i.e. the soil was broken into aggregates, packed into cores and equilibrated to −5 kPa (REPACKED). A flooded treatment (puddled soil remained flooded until harvest) was set as a control (FLOODED). Soil bulk density, penetration resistance and X-ray Computed Tomography (CT) derived macropore structure were measured. Total root length, root surface area, root volume, average diameter, and tip number were determined by WinRhizo.

**Results:**

AWD induced formation of macropores and slightly increased soil mechanical impedance. The total root length of the AWD and REPACKED treatments were 1.7–2.2 and 3.5–4.2 times greater than that of the FLOODED treatment. There was no significant difference between WET and DRY-WET treatments. The differences between genotypes were minimal.

**Conclusions:**

AWD influenced soil physical properties and some root characteristics of rice seedlings, but drying soil initially to −50 kPa versus −5 kPa had no impact. Macropores formed intentionally from repacking caused a large change in root characteristics.

## Introduction

Rice (*Oryza sativa* L.) is the staple food for over half of the world’s population (Chen et al. 2014). About 75% of total rice productivity comes from irrigated lowland rice systems (Bouman and Tuong 2001) that consume an estimated 24%–30% of the world’s developed freshwater resources (Bouman et al. 2007). This is a major sustainability challenge (Bouman and Tuong 2001) as water scarcity threatens the productivity of irrigated rice systems (Bouman et al. 2005). As a solution, alternate wetting and drying (AWD) is gaining adoption to decrease water demands, with large-scale international projects by the International Rice Research Institute (IRRI) and others promoting this technology (Lampayan et al. 2015).

AWD is likely to produce different soil physical conditions for rice growth than a flooded system, potentially influencing cultivar choice to maximise plant performance. Drying and wetting cycles from AWD have been shown to affect paddy soil structure compared to flooded systems (Zhang et al. 2003) and it has been demonstrated that AWD irreversibly increases soil strength at least in the top 12 cm of the soil (Norton et al. 2017). Puddled and flooded rice soils have little strength and much of the soil structure has been broken apart by mechanical action (Liu et al. 2005; Ringrose-Voase et al. 2000). Drying by AWD consolidates and shrinks the soil. Yoshida and Hallett (2008) found drying of paddy soils to −50 kPa water potential increased mechanical strength considerably, and that this strength did not decrease with subsequent wetting. Macropores may form as cracks and pre-existing pores that extend (Bottinelli et al. 2016), creating connected pore systems favourable to rapid root growth. Under AWD, roots may therefore experience greater mechanical impedance from the soil matrix, but take greater advantage of newly formed pore networks. Root elongation of cereal crops is strongly influenced by physical properties (Bengough et al. 2011; Valentine et al. 2012; White and Kirkegaard 2010). Cairns et al. (2004) found that the increase of penetration resistance induced by drying potentially limits the growth of new rice nodal roots. However, the presence of macropores may offset the effect of mechanical impedance. In an arable, upland farming system, Lampurlanés and Cantero-Martinez (2003) found that greater soil strength under no-tillage does not greatly affect root growth in well-structured soils.

Zhang et al. (2009) concluded moderate AWD (re-watered when soil water potential reached −15 kPa) can enhance rice root growth and improve grain yield, while a severe AWD (re-watered when soil water potential reached −30 kPa) limits rice root growth and decreases grain yield. These results were also reflected in a recent meta-analysis of 56 studies on the impacts of AWD on yield, it was observed that mild AWD (≥ − 20 kPa) did not cause a significant decrease in yield, however under AWD when water potential was less than −20 kPa a significant decrease in yield was observed (Carrijo et al. 2017). Perhaps −20 kPa drying produced highly restrictive root growth conditions. Monshausen and Gilroy (2009) found that mechanical stimulation of roots (i.e. transient bending) could elicit lateral root formation, possibly contributing to the positive impact of AWD at −15 kPa drying found by Zhang et al. (2009). The response of rice roots to AWD of paddy soil is still poorly understood and merits greater research interest.

Many reports have shown that root system architecture is influenced by both the soil environment and genotype (Acuña et al. 2007; Rogers et al. 2016). Rice genotypes with deeper roots are selected to improve resource capture under water saving irrigation strategies, such as AWD (Fang et al. 2013; Trachsel et al. 2011; Venuprasad et al. 2011), whereas shallow roots capture phosphorous more effectively (Clark et al. 2011). Breeding for root system architectures to maximize soil exploration and plant fitness (McCully 1995) offers considerable potential to improve yields, but too little thought has been given to root system response to soil physical properties (McKenzie et al. 2009). Roots may also induce changes to soil pore structure. By penetrating the soil, roots form macropores and create weak zones that are easy to fragment (Angers and Caron 1998). In AWD, surface cracks can be evident and the soil pore structure changes with drying (Ringrose-Voase et al. 2000).

The aim of this study was to explore the response of two contrasting rice genotypes with contrasting root architecture to soil physical conditions induced by hydraulic stress history or a manipulated macropore structure. To our knowledge, this is the first study to explore the interaction between soil strength, pore structure and rice genotype on rice root growth, with the results relevant to crop selection and soil management in AWD systems. For the AWD treatments the soil was either maintained in a puddled state, equilibrated to a constant water potential (−5 kPa water potential), or dried (−50 kPa water potential) and then rewetted (−5 kPa water potential). Our AWD simulates an initial drying cycle, with young plants studied so that cores of a suitable size for X-ray Computed Tomography (CT) could be used with minimal confinement of root growth. In AWD a water potential of −5 kPa will typically occur before water reaches a 15 to 20 cm depth where subsequent flooding is recommended (Yang et al. 2017). A water potential of −50 kPa is more extreme, above the thresholds of −10 to −20 kPa water potential where adverse effects on plant growth stages can occur, but often achieved in the field during periods of high plant transpiration and hydraulic gradients from the soil surface to the water table. Soil physical conditions were characterised from core measurements of bulk density and penetration resistance, and a detailed X-ray CT analysis of macropore structure. We compared two rice genotypes, shallow rooting IR64 and deeper rooting Black Gora. Our hypothesis was that soil mechanical impedance to root growth in a paddy system would be dictated by the greatest drying stress, with differing impacts on the root morphology of rice seedlings between deep- and shallow-rooting genotypes. Some of these differences would be due to the creation of macropores, which would provide preferential channels for root growth that would overcome limitations from soil strength. The research has relevance to developing screening approaches of rice genotypes specifically for AWD systems. Moreover, it provides new information on how AWD may influence soil physical conditions.

## Material and methods

### Rice cultivars and soil properties

Contrasting rice genotypes were used in the study: IR 64 (an indica type with a shallow root system from the Oryza SNP set (McNally et al. 2009)) and Black Gora (an aus type with deep root system from the Rice Diversity Panel 1 (Zhao et al. 2011)). The soil used in the experiment was sampled from a paddy field maintained as permanent rice by the CREA Unità di Ricerca per la Risicoltura in Vercelli, Italy and located at 45°19′25” N and 8°22′25″ E. In the top 20 cm the soil texture consisted of 61% sand, 26% silt and 13% clay determined by the combination of wet sieving and hydrometer methods. It has 2.5% organic carbon measured with a CNS elemental analyser (CE Instruments, Wigan, UK) and pH of 6.7 measured in a 1:5 soil to CaCl_2_ using a pH meter (Hanna Instruments, Leighton Buzzard, UK).

### Experimental design and growth conditions

We used PVC soil cores that were 5 cm in diameter and 8 cm high chosen to obtain an X-ray CT resolution <40 μm so that macropores could be resolved. The cores were filled with soil that had been puddled by stirring soil and water thoroughly to mimic a paddy field. Four treatments were established: (1) FLOODED: puddled soil which remained flooded until harvest; (2) WET: puddled soil which was dried and maintained at −5 kPa to simulate the wet end of AWD; (3) DRY-WET: Puddled soil first equilibrated to −50 kPa followed by flooding and drying to −5 kPa to simulate a more drastic AWD cycle; and (4) REPACKED: Puddled soil that was first equilibrated to −50 kPa then broken into aggregates smaller than 4 mm before packing into cores to a similar bulk density to FLOODED, then flooded and dried to −5 kPa. A suction plate with a bubbling pressure of −75 kPa was used to equilibrate water potential (Ecotech, Bonn, Germany). Each treatment had four replicates.

Each core was planted with one rice seedling. The seeds were germinated on wet filter paper at 30 °C for 48 h before being planted 3 mm below the soil surface. Plants were grown in a heated greenhouse with day/night temperatures of 28/26 °C and an 11 h photoperiod. The WET, DRY-WET and REPACKED treatments were grown on a large sand table that maintained the water potential. Over the first 3 days, the water potential was kept at −0.5 kPa to decrease stress on young seedlings, and then changed to −5 kPa until harvest. The FLOODED treatment cores were placed in a plastic tray filled with water to keep the cores flooded during the whole growth period. Plastic beads were put on the surface of cores to reduce evaporation. Rice cultivars were grown for 14 days. Each pot was irrigated daily by adding 10 ml of water to the top to compensate for the evaporative losses. This was quickly drained by the sand table for the WET, DRY-WET and REPACKED treatments to the prescribed water potential, but the hydraulic gradient in the core from evaporation may have induced a more negative water potential at the soil surface before watering.

### Penetration resistance and bulk density measurements

Before planting the rice, penetration resistance was measured by a Z005 mechanical test frame fitted with a 5 N load cell accurate to 0.05 mN (Zwick/Roell AG, Ulm, Germany). A 1 mm diameter, 30° full opening angle miniature cone penetrometer was inserted into the cores to a depth of 4 mm at a speed of 2 mm min^−1^. Three points were measured for every core. We defined soil penetration resistance as the plateau in the penetration stress measured during penetration. At the same time the bulk density was calculated from the mass of dry soil and volume.

### X-ray computed tomography and image processing

Soil cores were scanned using a XT H 225 ST CT scanner (Nikon Metrology, Tring, UK) with settings of 180 kV, 285 μA, 0.12° steps with 500 ms exposure time, 0.5 mm Cu filter and pixel size at 43.09 μm. Shortly before scanning, all the shoots were cut to slow root growth. All the cores were then drained to −50 kPa on the suction table to improve the quality of the CT images (Zappala et al. 2013). Drainage likely induces shrinkage in the FLOODED and WET treatments, which will be considered when interpreting the results. Drainage of pore water and storage after scanning was undertaken at 4 °C to avoid decomposition of roots until root-washing.

Three dimensional reconstruction was performed on the original images using the software CT Pro 3D (Nikon Metrology, version XT 4.3.1). The digital image processing and analysis were conducted with ImageJ (Version 1.50e). The 3D image stack of each soil core column was cropped to a region of interest of 600 × 600 pixels (25.85 × 25.85 mm) and a depth of 800 continuous slices (34.47 mm). Cropping the images and reducing the stacks was necessary to avoid ring artefacts caused by edge effects and beam hardening (Deurer et al. 2009; Mooney et al. 2006). Images were segmented using a ‘Default’ thresholding method, a variation on the ‘IsoData’ method where the average of the object and background image are used to compute the threshold (Ridler and Calvard 1978). Porosity and pore size distribution were computed using the ‘thickness’ plugin of ‘BoneJ’. This approach fits the largest sphere inside the 3D pore space that touches the bordering soil matrix and then measures the sphere diameter.

Unfortunately the moisture content of the soil created considerable overlap between the greyscale values for the roots and the adjacent water filled pore space. This, combined with the small diameter of the rice roots (<0.1 mm), meant that it was not possible to accurately segment the roots in the CT images in this study.

### Root traits

After CT scanning, roots were carefully washed from the soil. Roots with soil were placed on a sieve (0.5 mm) and carefully washed with tap water to remove all soil particles. Root samples were placed in a plexiglas tray (100 by 200 mm) with a 4 to 6 mm deep layer of water, and spread out with tweezers to minimize overlapping. Grayscale images (800 DPI) of roots were obtained using an Expression 10000XL scanner (Epson, Suwa, Japan). Total root length, root surface area, root volume, average diameter, and tip number were determined by root analysis software, WinRhizo (Version 2013e) (Regent Instrument Canada Inc.). If not scanned immediately, the roots were immersed in a 50% ethanol solution in plastic containers with lids, and stored at 4 °C. On a subset of cores, manual counts of root tips were performed to check the accuracy of WinRhizo.

### Statistical analysis

Data were checked for normality with probability plots. One­way ANOVA and post hoc analysis were conducted by the Fisher’s protected least significant difference (LSD) procedure with SPSS 24.0 to evaluate for significant differences between treatments (*P* ≤ 0.01). Significant statistical differences of pore size distribution between rice cultivars were established by the Students t-test.

## Results

### Soil physical conditions

**Fig. 1:**
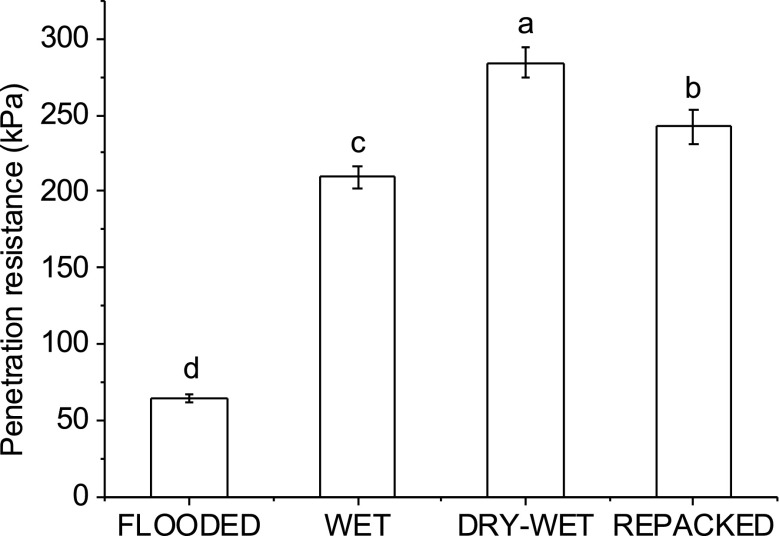
Penetration resistance of four treatments before planting rice. Error bars are standard error of the mean. Different letters above bars indicate that the means are significantly different (*P* < 0.01) (*n* = 30)

There was no significant difference between rice cultivars for the initial soil physical properties and growing conditions. The soil of the FLOODED treatment was the wettest and weakest, and its penetration resistance was 69.2%–77.3% less than the other treatments (Fig. 1). Penetration resistance of the DRY-WET treatment was 35.7% greater than that of the WET treatment (Fig. 1). This was in agreement with our hypotheses that soils dried to −50 kPa and then rewet to −5 kPa would be stronger than soils maintained at −5 kPa. For the REPACKED treatment, the penetration resistance was ranked between the WET treatment and the DRY-WET treatment (Fig. 1).

A small 2.4% increase in bulk density was caused by shrinkage of the WET and DRY-WET treatments, compared to FLOODED and REPACKED soils that had similar bulk densities (*P* < 0.05) (Table 1). This was reflected in the calculated total porosity, but when separated into air-filled porosity at −5 kPa, the equivalent of 60 μm macropores, the REPACKED cores were very different to the FLOODED ones. In the DRY-WET treatment, visible cracks were created when dried to −50 kPa, as detected by the greater air-filled porosity compared to the WET treatment. Although the water potentials during the growing period were the same for the treatments, except for the FLOODED treatment, the water contents were different because their different soil structures affected their water holding capacity (Table 1).

**Table 1:** Selected physical properties of soils of four treatments. Numbers in brackets are standard deviation of the mean

Treatments	Bulk density (g cm^−3^)	Total porosity (m^3^ m^−3^)	Air-filled porosity (m^3^ m^−3^)	Macroporosity from CT images (>43 μm)	Volume water content during rice growth (cm^3^ cm^−3^)	The greatest water potential history (kPa)	Water potential during rice growth (kPa)
FLOODED	1.472(0.004)b	0.444(0.001)a	0.001(0.001)d	NA	0.443(0.002)a	0	0
WET	1.512(0.006)a	0.430(0.002)b	0.028(0.003)c	0.014(0.002)b	0.402(0.001)b	−5	−5
DRY-WET	1.513(0.011)a	0.429(0.004)b	0.038(0.007)b	0.015(0.001)b	0.391(0.004)c	−50	−5
REPACKED	1.476(0.003)b	0.443(0.001)a	0.119(0.004)a	0.131(0.009)a	0.324(0.004)d	−50	−5

Different letters indicate that the means are significantly different (*P* < 0.01). NA means not available

### Macroporosity structure from X-ray CT images

**Fig. 2:**
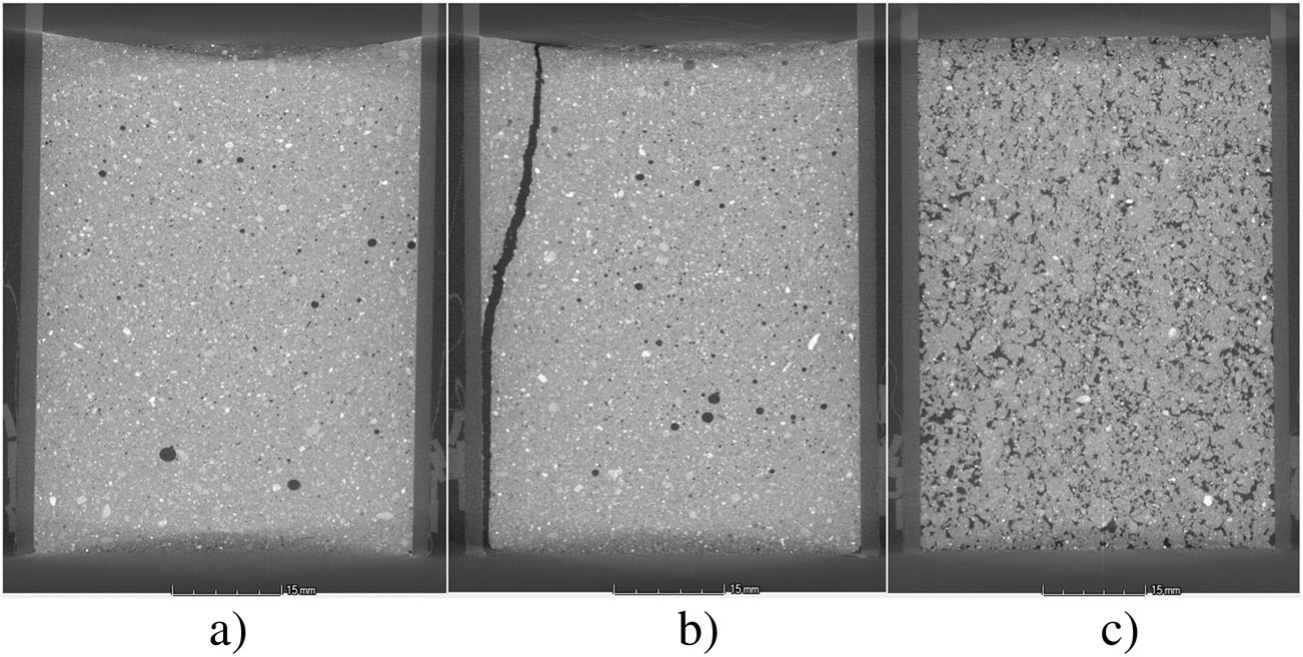
2D greyscale images of soil cores from X-ray CT before planting rice. **a** WET treatment; **b** DRY-WET treatment; **c** REPACKED treatment. The plastic wall of the 5 cm diameter soil core is visible

**Fig. 3:**
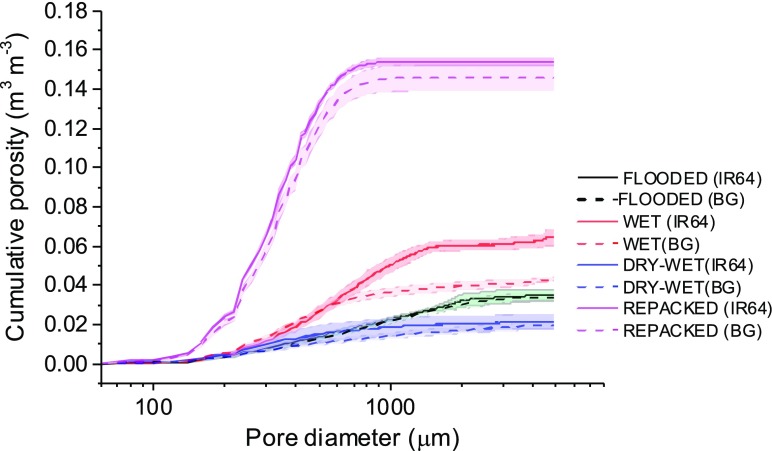
Cumulative porosity of soils after harvest. The shaded area around the lines is the standard error of the means

Cross-sections of the cores before plant growth are shown in Fig. 2. After plant growth, harvest and drying to −50 kPa, the total cumulative macroporosity (>43 μm) and pore size distribution obtained by CT showed differences between treatments (Fig. 3). For the REPACKED treatment, the macroporosity of both IR64 and Black Gora was much greater than other treatments (*P* < 0.01), with no difference between cultivars (Fig. 2). For the WET treatment, the macroporosity of IR64 was 47.0% more than Black Gora and their pore size distribution also showed significant differences (*P* < 0.01). Visual examination showed this was caused by pores >500 μm, with IR64 having 128% greater pore volume in this size range than Black Gora (Fig. 3). The macroporosity of FLOODED and DRY-WET treatments was less than the other two treatments and porosity of pores in each size class (43–4900 μm) was also less than the other two treatments. Pore size distribution of IR64 and Black Gora were not statistically different for either the FLOODED and DRY-WET treatments based on t-tests at a range of pores sizes (Fig. 3).

### Root traits

**Fig. 4:**
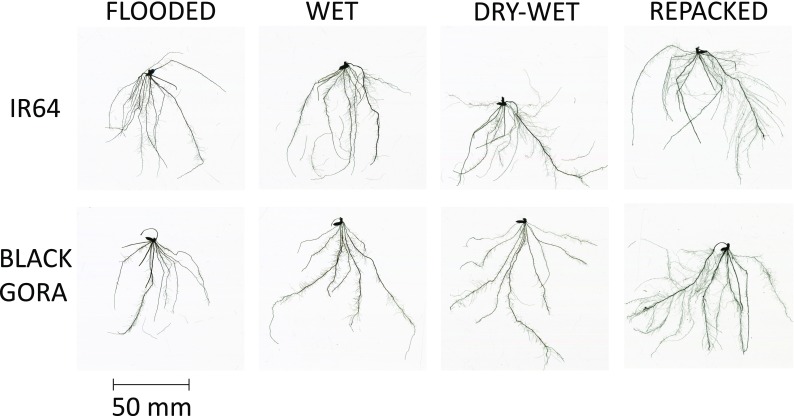
2D root images. **a** IR64, FLOODED treatment; **b** IR64, WET treatment; **c** IR64, DRY-WET treatment; **d** IR64, REPACKED treatment; **e** Black Gora, FLOODED treatment; **f** Black Gora, WET treatment; **g** Black Gora, DRY-WET treatment; **h** Black Gora, REPACKED treatment

Images of the different root architectures between treatments are shown in Fig. 4. For all the root parameters, only the average diameter of the root system was significantly different between genotypes. The diameter of Black Gora was 20.6% greater than that of IR64 in the FLOODED treatment and was 10.8% less than that of IR64 in WET treatment (Table 2). When comparing the differences between soil treatments for the same genotype, they followed the same trend. The diameter of the REPACKED treatment was 16.1%–22.1% less than other treatments for IR64 and 14.4%–35.3% less than other treatments for Black Gora. The diameter of other three treatments were not significantly different (Table 2).

**Table 2:** Root traits and stem mass of two rice genotypes. Numbers in brackets are standard deviation of the mean

Genotypes	Treatments	Total root length (cm)	Root diameter (mm)	Surface area (cm^2^)	Root volume (cm^3^)	Number of tips	Shoot mass (g)	Root mass (g)
IR64	FLOODED	204(43)Ac	0.155(0.016)Ba	8.27(2.1)Ac	0.053(0.008)Ac	539(241)Ac	0.026(0.004)Ab	0.008(0.003)Aa
	WET	351(68)Ab	0.167(0.013)Aa	14.6(2.4)Ab	0.097(0.015)Ab	1018(158)Ab	0.033(0.002)Aa	0.012(0.003)Aa
	DRY-WET	349(74)Ab	0.158(0.017)Aa	13.1(1.0)Ab	0.085(0.008)Ab	1181(82)Ab	0.029(0.005)Aab	0.011(0.002)Aa
	REPACKED	708(49)Aa	0.130(0.014)Ab	21.3(2.5)Aa	0.130(0.017)Aa	2354(225)Aa	0.030(0.003)Bab	0.010(0.001)Aa
Black Gora	FLOODED	162(51)Ac	0.187(0.009)Aa	7.57(2.1)Ac	0.060(0.020)Ac	403(111)Ac	0.031(0.009)Aa	0.008(0.002)Ab
	WET	363(58)Ab	0.149(0.006)Bb	13.9(1.9)Ab	0.091(0.015)Aab	1092(264)Ab	0.036(0.013)Aa	0.012(0.002)Aa
	DRY-WET	341(112)Ab	0.153(0.007)Ab	13.3(4.4)Ab	0.084(0.029)Abc	1145(74)Ab	0.031(0.009)Aa	0.011(0.002)Aa
	REPACKED	690(101)Aa	0.121(0.010)Ac	20.9(2.9)Aa	0.122(0.020)Aa	2149(261)Aa	0.043(0.004)Aa	0.011(0.001)Aa

Different capital letters indicate that the means of genotypes of the same treatment are significantly different (*P* < 0.01). Different lowercases indicate that the means of treatments of one genotype are significantly different (*P* < 0.01)

For both IR64 and Black Gora, total root length, surface area, root volume and number of tips of the REPACKED treatment were significantly greater than other treatments (*P* < 0.01) (Table 2). Specifically, the total root length of the REPACKED treatment was 3.47 times greater than that of the FLOODED treatment and c. 2 times greater than the WET and DRY-WET treatments for IR64. For Black Gora, the total root length of the REPACKED treatment was 4.26 times greater than that of FLOODED treatment and c. 2 times greater than that of WET and DRY-WET treatments (Table 2). Surface area, root volume and the number of root tips followed the same trend with total root length, i.e. REPACKED > WET and DRY-WET > FLOODED. The difference between treatments for number of tips was greater than that of other root parameters. For IR64, the number of tips of the REPACKED treatment was 4.37 times greater than that of the FLOODED treatment and 1.99–2.31 times greater than that of the WET and DRY-WET treatment. For Black Gora, the number of tips of the REPACKED treatment was 5.33 times greater than that of the FLOODED treatment and 1.88–1.97 times greater than the WET and DRY-WET treatment (Table 2). The root mass of different soil treatments did not show significant differences for IR64, while for Black Gora, the root mass of FLOODED treatment was 27.3%–33.3% less than other treatments (Table 2). The shoot mass of the WET treatment was 26.9% greater than the FLOODED treatment for IR64, while for Black Gora, it was not affected by AWD or soil packing. In addition, the shoot mass of Black Gora was 43.3% greater than that of IR64 for the REPACKED treatment (Table 2).

## Discussion

The hypothesis that root morphology would vary due to the severity of AWD was not confirmed in this study. In rice production systems, AWD usually re-floods rice when it is wetter than −50 kPa water potential, the driest water potential that we used (Belder et al. 2004; Bouman et al. 2007; Norton et al. 2017). However, the hydraulic gradient to the evaporating surface and spatial heterogeneity of soil physical properties in the field (Becel et al. 2012; Valentine et al. 2012) could impart even greater hydraulic stresses on the soil. We found that soil drying caused an irreversible change to penetration resistance upon rewetting, albeit with mechanical impedance levels not limiting to root growth (Bengough et al. 2011). Root growth and AWD did induce the formation of macropores, and in comparision to the FLOODED treatment, this may be one cause of differences in root architecture. When macroporosity was intentionally manipulated in the REPACKED treatment, there were huge differences in root morphology. This treatment, with a prominence of interaggregate macropores, promoted root elongation and branching (Table 2).

Despite observing large differences in soil physical condition between the FLOODED, WET, DRY-WET and REPACKED treatments, the contrasting deep rooting Black Gora and shallow rooting IR64 genotypes generally did not differ markedly in either root structure or their impact on soil macroporosity. Between these genotypes, the only plant phenotypic difference was slightly greater average root diameter (12%) for IR64. To enable X-ray CT imaging we limited the study to small cores and young plants, but the root traits of seedlings may not be indicative of older plants (Atkinson et al. 2014), so follow-on work with larger cores is necessary. Moreover, in an unsuccessful attempt to resolve rice roots in X-Ray CT imaging, all soils were dried to −50 kPa before final scanning to improve segmentation (Zappala et al. 2013). This will inevitably induce shrinkage and crack formation (Yoshida and Hallett 2008), particularly in the WET and FLOODED treatments that never experienced −50 kPa during plant growth. With drying to −50 kPa the combination of the presence of roots and the shrinkage stress could dissipate macropore formation to a greater number of smaller pores. This was particularly evident for the WET treatment. In the DRY-WET treatment, shrinkage to −50 kPa before plant growth likely consolidated the soil, with only a few large shrinkage cracks forming near the sample edge (Fig. 2) that were outside the analysed volume.

An interesting finding for AWD systems was the formation of macropores and their potential to influence root morphology. Macropores could provide rapid root growth pathways in soil, and on re-wetting AWD systems they could improve water permeability to soil in the rooting zone above confining plough pans that are common in paddy rice systems. Passioura (1991) hypothesised that roots are not evenly distributed throughout the soil matrix and are possibly trapped in large pores. The hypothesis has been verified by many subsequent studies. Colombi et al. (2017) created artificial, vertical macropores in the soil and observed via X-ray imaging that roots of wheat, soybean and maize can grow preferentially towards these macropores, although they may choose to cross through them rather than penetrate through them. Pfeifer et al. (2014) also found from X-ray imaging that barley roots tended to grow towards macorpores in compacted soils. White and Kirkegaard (2010) observed that wheat roots preferred to grow in pores and structural cracks in dense, structured soil below 0.6 m.

Root elongation remains relatively unimpeded as long as the root tip remains “trapped” in the macropore (Pierret et al. 2007). Pierret et al. (1999) grew wheat plants in undisturbed soil cores from the field and in repacked soil cores filled at the same bulk density and found plants grew better in repacked cores than in undisturbed cores where 80% of roots were located in macropores. They also found no difference between macropore sheath and bulk soil for both bacterial population and elements concentrations in repacked soil. This suggests that our REPACKED treatment might provide a good physical environment for roots, rather than biochemistry driving differences.

Mechanical impedance is one of the major limitations for root system growth and development (Bengough et al. 2011). The penetration resistance between our different soil treatments was far below the critical threshold of 2 MPa (Ringrose-Voase et al. 2000). However, a negative relationship between root elongation rate and penetration resistance has been observed for weaker soils (Bengough et al. 2011; Thangaraj et al. 1990). Whitmore and Whalley (2009) proposed root elongation rate decreases almost linearly with the increase of penetration resistance up to critical levels where elongation may cease. Our study did not find a negative relationship between root length (i.e. elongation) and penetration resistance. Whilst the penetration resistance of the AWD and REPACKED treatments was much greater than that of FLOODED treatment, the root length of the FLOODED treatment was much less than that of other treatments (Fig. 1 & Table 2). Others have observed a poor correlation between soil strength and rice root elongation in weaker soils (Rogers et al. 2016).

The greatest impact on root growth that we observed was the influence of macropores in the REPACKED treatment. Despite the REPACKED treatment having a penetration resistance that was closer to the WET and DRY-WET soil treatments than the flooded treatment, the REPACKED treatment had the largest root mass and length of all. In structured soils this suggests that mechanical impedance measurements obtained by rigid penetrometers could be of limited use (Bengough and Mullins 1990). Although a penetrometer is a direct and easy way to measure penetration resistance, measurements need to be interpreted with associated information on pore structure to assess restrictions to root growth with greater rigour. This is supported by a study on spring wheat by Gaiser et al. (2013) who found that soil penetration resistance became much less significant for spring wheat root growth above biopore volumes between 0.015 and 0.030 m^3^ m^−3^.

Soil structure affects a range of physical limitations to root growth, including water, air and mechanical impedance (Whitmore and Whalley 2009). Mechanical impedance has been found to have a larger impact than water stress during drought (volumetric water content was 17%–24%) on rice root growth (Cairns et al. 2004). In a broad field survey of physical limitation to barley root growth, Valentine et al. (2012) found that the volume of pores between 60 μm and 300 μm equivalent diameter (estimated from water-release characteristics) accounted for 65.7% of the variation in root elongation rates. We observed that greater drying by AWD increased both mechanical impedance and macropore development, with recovery not found with subsequent flooding. Consequently, root morphology was also altered, but not following expected trends for penetration or bulk density differences. Bulk density is a widely used parameter to quantify soil compaction, but it is poor at describing soil functions like the rate of root growth (Colombi et al. 2017). In our study, the bulk density of the four soil treatments was almost the same, but closer examination of soil macropores found large differences that may explain observed root morphology differences. Simple measurements like penetration resistance and bulk density provide an incomplete description of physical stresses experienced by growing roots, suggesting that macropores should not be neglected. There is great potential with non-invasive imaging to study these processes in much greater detail, including in naturally structured soils.

## Conclusions

In comparison to the puddled state of paddy rice systems, the hydraulic stress induced by drying similar to the first cycle of AWD increased many root traits that are important to plant productivity. Imparting mild drying stresses of −5 kPa or − 50 kPa increased penetration resistance by more than 400% compared to puddled soil, with subsequent rewetting having minimal impact on soil strength. The increased root tips after a hydraulic stress was imposed may be due to branching induced by mechanical impedance or the development of macropores that serve as preferential root growth pathways. Further investigations with REPACKED cores containing a large volume of macropores found an even greater impact to root traits than AWD. A comparison between contrasting deep-rooting (Black Gora) and shallow-rooting (IR64) rice genotypes found little cultivar specific impact of the soil physical properties to root traits, or of the roots to soil physical properties. Further research should explore more mature plants and tracking the interaction between soil strength, pore structure development with AWD and rice genotypes. There may be potential in rice cultivation systems to manipulate soil structure through either tillage, cycles of wetting and drying or structure forming amendments like organic residues to enhance root structure. Simple measurements of soil physical properties such as bulk density or penetration resistance, as to their effects on root growth alone, may provide an incomplete assessment. A greater emphasis on the properties of macropores that provide easier growth pathways for roots is needed. Future research should also explore root phenotypic traits that may improve root: soil interactions in mechanically constrained soils where macropores provide important growth pathways.

## References

[CR1] Acuña TB, Pasuquin E, Wade L (2007). Genotypic differences in root penetration ability of wheat through thin wax layers in contrasting water regimes and in the field. Plant Soil.

[CR2] Angers DA, Caron J (1998) Plant-induced changes in soil structure: processes and feedbacks. In: Plant-induced soil changes: processes and feedbacks. Springer, pp 55–72

[CR3] Atkinson JA, Rasmussen A, Traini R, Voss U, Sturrock C, Mooney SJ, Wells DM, Bennett MJ (2014). Branching out in roots: uncovering form, function, and regulation. Plant Physiol.

[CR4] Becel C, Vercambre G, Pages L (2012). Soil penetration resistance, a suitable soil property to account for variations in root elongation and branching. Plant Soil.

[CR5] Belder P, Bouman B, Cabangon R, Guoan L, Quilang E, Yuanhua L, Spiertz J, Tuong T (2004). Effect of water-saving irrigation on rice yield and water use in typical lowland conditions in Asia. Agric Water Manag.

[CR6] Bengough AG, Mullins CE (1990). Mechanical impedance to root growth: a review of experimental techniques and root growth responses. Eur J Soil Sci.

[CR7] Bengough AG, McKenzie B, Hallett P, Valentine T (2011). Root elongation, water stress, and mechanical impedance: a review of limiting stresses and beneficial root tip traits. J Exp Bot.

[CR8] Bottinelli N, Zhou H, Boivin P, Zhang Z, Jouquet P, Hartmann C, Peng X (2016). Macropores generated during shrinkage in two paddy soils using X-ray micro-computed tomography. Geoderma.

[CR9] Bouman B, Tuong TP (2001). Field water management to save water and increase its productivity in irrigated lowland rice. Agric Water Manag.

[CR10] Bouman B, Peng S, Castaneda A, Visperas R (2005). Yield and water use of irrigated tropical aerobic rice systems. Agric Water Manag.

[CR11] Bouman B, Feng L, Tuong T, Lu G, Wang H, Feng Y (2007). Exploring options to grow rice using less water in northern China using a modelling approach: II. Quantifying yield, water balance components, and water productivity. Agric Water Manag.

[CR12] Cairns JE, Audebert A, Townend J, Price AH, Mullins CE (2004). Effect of soil mechanical impedance on root growth of two rice varieties under field drought stress. Plant Soil.

[CR13] Carrijo DR, Lundy ME, Linquist BA (2017). Rice yields and water use under alternate wetting and drying irrigation: a meta-analysis. Field Crop Res.

[CR14] Chen H, Xie W, He H, Yu H, Chen W, Li J, Yu R, Yao Y, Zhang W, He Y (2014). A high-density SNP genotyping array for rice biology and molecular breeding. Mol Plant.

[CR15] Clark RT, MacCurdy RB, Jung JK, Shaff JE, McCouch SR, Aneshansley DJ, Kochian LV (2011). Three-dimensional root phenotyping with a novel imaging and software platform. Plant Physiol.

[CR16] Colombi T, Braun S, Keller T, Walter A (2017). Artificial macropores attract crop roots and enhance plant productivity on compacted soils. Sci Total Environ.

[CR17] Deurer M, Grinev D, Young I, Clothier BE, Mueller K (2009). The impact of soil carbon management on soil macropore structure: a comparison of two apple orchard systems in New Zealand. Eur J Soil Sci.

[CR18] Fang S, Clark RT, Zheng Y, Iyer-Pascuzzi AS, Weitz JS, Kochian LV, Edelsbrunner H, Liao H, Benfey PN (2013). Genotypic recognition and spatial responses by rice roots. Proc Natl Acad Sci U S A.

[CR19] Gaiser T, Perkons U, Küpper PM, Kautz T, Uteau-Puschmann D, Ewert F, Enders A, Krauss G (2013). Modeling biopore effects on root growth and biomass production on soils with pronounced sub-soil clay accumulation. Ecol Model.

[CR20] Lampayan RM, Rejesus RM, Singleton GR, Bouman BA (2015). Adoption and economics of alternate wetting and drying water management for irrigated lowland rice. Field Crop Res.

[CR21] Lampurlanés J, Cantero-Martinez C (2003). Soil bulk density and penetration resistance under different tillage and crop management systems and their relationship with barley root growth. Agron J.

[CR22] Liu C, Yu W, Chen W, Chen S (2005). Laboratory investigation of plough sole reformation in a simulated paddy field. J Irrig Drain Eng.

[CR23] McCully M (1995). Water efflux from the surface of field-grown grass roots. Observations by cryo-scanning electron microscopy. Physiol Plantarum.

[CR24] McKenzie BM, Bengough AG, Hallett PD, Thomas W, Forster B, McNicol J (2009). Deep rooting and drought screening of cereal crops: a novel field-based method and its application. Field Crop Res.

[CR25] McNally KL, Childs KL, Bohnert R, Davidson RM, Zhao K, Ulat VJ, Zeller G, Clark RM, Hoen DR, Bureau TE, Stokowski R, Ballinger DG, Frazer KA, Cox DR, Padhukasahasram B, Bustamante CD, Weigel D, Mackill DJ, Bruskiewich RM, Ratsch G, Buell CR, Leung H, Leach JE (2009). Genomewide SNP variation reveals relationships among landraces and modern varieties of rice. Proc Natl Acad Sci U S A.

[CR26] Monshausen GB, Gilroy S (2009). The exploring root—root growth responses to local environmental conditions. Curr Opin Plant Biol.

[CR27] Mooney SJ, Morris C, Berry PM (2006). Visualization and quantification of the effects of cereal root lodging on three-dimensional soil macrostructure using X-ray computed tomography. Soil Sci.

[CR28] Norton GJ, Shafaei M, Travis AJ, Deacon CM, Danku J, Pond D, Cochrane N, Lockhart K, Salt D, Zhang H (2017). Impact of alternate wetting and drying on rice physiology, grain production, and grain quality. Field Crop Res.

[CR29] Passioura J (1991). Soil structure and plant-growth. Aust J Soil Res.

[CR30] Pfeifer J, Kirchgessner N, Walter A (2014). Artificial pores attract barley roots and can reduce artifacts of pot experiments. J Plant Nutri Soil Sci.

[CR31] Pierret A, Moran C, Pankhurst C (1999). Differentiation of soil properties related to the spatial association of wheat roots and soil macropores. Plant Soil.

[CR32] Pierret A, Doussan C, Capowiez Y, Bastardie F (2007). Root functional architecture: a framework for modeling the interplay between roots and soil. Vadose Zone J.

[CR33] Ridler TW, Calvard S (1978). Picture thresholding using an iterative selection method. IEEE Trans Syst Man Cybern.

[CR34] Ringrose-Voase A, Kirby J, Djoyowasito G, Sanidad W, Serrano C, Lando TM (2000). Changes to the physical properties of soils puddled for rice during drying. Soil Till Res.

[CR35] Rogers ED, Monaenkova D, Mijar M, Nori A, Goldman DI, Benfey PN (2016). X-ray computed tomography reveals the response of root system architecture to soil texture. Plant Physiol.

[CR36] Thangaraj M, O'toole J, De Datta S (1990). Root response to water stress in rainfed lowland rice. Exp Agric.

[CR37] Trachsel S, Kaeppler SM, Brown KM, Lynch JP (2011). Shovelomics: high throughput phenotyping of maize (*Zea mays* L.) root architecture in the field. Plant Soil.

[CR38] Valentine TA, Hallett PD, Binnie K, Young MW, Squire GR, Hawes C, Bengough AG (2012). Soil strength and macropore volume limit root elongation rates in many UK agricultural soils. Ann Bot.

[CR39] Venuprasad R, Impa S, Gowda RV, Atlin G, Serraj R (2011). Rice near-isogenic-lines (NILs) contrasting for grain yield under lowland drought stress. Field Crop Res.

[CR40] White RG, Kirkegaard JA (2010). The distribution and abundance of wheat roots in a dense, structured subsoil–implications for water uptake. Plant Cell Environ.

[CR41] Whitmore AP, Whalley WR (2009). Physical effects of soil drying on roots and crop growth. J Exp Bot.

[CR42] Yang J, Zhou Q, Zhang J (2017). Moderate wetting and drying increases rice yield and reduces water use, grain arsenic level, and methane emission. Crop J.

[CR43] Yoshida S, Hallett P (2008). Impact of hydraulic suction history on crack growth mechanics in soil. Water Resour Res.

[CR44] Zappala S, Mairhofer S, Tracy SR, Sturrock CJ, Bennett MJ, Pridmore T, Mooney SJ (2013). Quantifying the effect of soil moisture content on segmenting root system architecture in X-ray computed tomography images. Plant Soil.

[CR45] Zhang P, Li L, Pan G, Zhang J (2003). Influence of long-term fertilizer management on topsoil microbial biomass and genetic diversity of a paddy soil from the tai Lake region, China. Acta Ecol Sin.

[CR46] Zhang H, Xue Y, Wang Z, Yang J, Zhang J (2009). An alternate wetting and moderate soil drying regime improves root and shoot growth in rice. Crop Sci.

[CR47] Zhao K, Tung CW, Eizenga GC, Wright MH, Ali ML, Price AH, Norton GJ, Islam MR, Reynolds A, Mezey J, McClung AM, Bustamante CD, McCouch SR (2011). Genome-wide association mapping reveals a rich genetic architecture of complex traits in Oryza sativa. Nat Commun.

